# A Modest Art: Securing Privacy in Technologically Mediated Homecare

**DOI:** 10.1007/s10699-015-9448-5

**Published:** 2015-12-14

**Authors:** Ike Kamphof

**Affiliations:** Faculty of Arts and Social Sciences, Maastricht University, Box 616, 6200 MD Maastricht, Netherlands

**Keywords:** Technomoral change, Reflection-in-action, Privacy, Art of living, Tele-monitoring

## Abstract

This article addresses the art of living in a technological culture as the active engagement with technomoral change. It argues that this engagement does not just take the form of overt deliberation. It shows in more modest ways as reflection-in-action, an experimental process in which new technology is fitted into existing practices. In this process challenged values are re-articulated in pragmatic solutions to the problem of working with new technology. This art of working with technology is also modest in the sense that it is not oriented to shaping one’s own subjectivity in relation to technology. It emanates from human existence as relational and aims at securing good relationships. The argument will be developed in relation to a case study of the ways in which homecare workers engaged with the value of privacy, challenged by tele-monitoring technology that was newly introduced into their work.

This essay investigates the art of living in a technological culture as the engagement with “technomoral change” (Swierstra 2013, p. 200). This engagement, I argue, does not just take place as overt deliberation on challenged values and the desirability of technologies. It shows in more modest ways in an everyday experimental tinkering to “fit” (Pols 2012, p. 25) new technology into practices. In this process challenged values are re-articulated in pragmatic solutions to ethical problems that surface in working with specific technologies. This modest ethical work can be seen as “reflection-in-action” (Schön 1983, p. 49), part of the “art” (p. 18) professionals bring to bear on indeterminate situations. This art of working with technology is also modest in the sense that it is not oriented to shaping one’s own subjectivity in relation to technology. It emanates from our existence as relational beings and is directed to securing good relationships. I develop my argument in relation to a case study of the ways in which caregivers in Dutch homecare engaged with the value of privacy, challenged by tele-monitoring technology for frail elderly people that was newly introduced into their work. Before I introduce the case and discuss its main characteristics as a process of reflection-in-action and as a relational activity, I will sketch the dynamics of technology and morality in a more general theoretical perspective.

## The Interplay of Technology and Morality

Though the social role of technology in changing how we live, work and communicate is widely acknowledged, attention to the ways in which technology specifically influences our morality is more recent (cf. Swierstra 2013; Verbeek 2011). Here I am interested in how new technologies can destabilize established moral values and lead to “technomoral change” (Swierstra 2013, p. 200). Building on a pragmatic view of morality, which I will follow, Swierstra sustains that acting morally in everyday life is largely a matter of practical routines. New technologies can break these routines and bring tacit values to awareness, which can lead to their (re)articulation and modification.

The relationship between technology and morality is not unidirectional. Obviously, values influence the development of new technologies. Moreover, technology users do not simply adjust their values to new technological possibilities that open up. What people believe in is bound up with their identity and the culture shared with others. Therefore, new options for perceiving the environment or for acting in it, offered by technology, can lead to moral conflict when technology pushes in one direction and established values in another. Pols (2012) demonstrates how much work healthcare professionals and clients invest to “fit” (p. 25) new technologies into daily care practices. Dealing with moral conflict and change is one aspect of fitting together technological applications, healthcare clients and care activities—new and existing ones.

Acknowledging that technology co-shapes morality thus does not exclude all belief in human agency—a necessary requirement to see people as moral beings in the first place. The question is what this agency looks like. Verbeek (2011) emphasizes the capability of people to develop a relationship to the technologies that influence them. Following Foucault, he speaks about this as a “self-practice” (p. 75), in which technology users “design” or “style” their subjectivity in interaction with technology. Swierstra (2013) calls for an active welcoming of opportunities for “moral innovation” (p. 215) and reflective engagement with technomoral change. In this essay I investigate a case of this kind of engagement. However, I will not follow Swierstra and Verbeek in their focus on overt deliberation and argument.

Swierstra (2013) speaks of the process of reflection, instigated by the disruptive impact of new technologies, as a process of stopping and thinking and discussing. He calls this “ethics” (p. 206) as opposed to a more tacit morality. However, in the same way as values in ordinary life are articulated in particular decisions and acts, reflective re-articulations of values can also take place within practice, in a process that Schön (1983) has called “reflection-in-action” (p. 21). Like Pols (2012), who speaks about the “fitting” activity of technology users in terms of a practical “modest aesthetics” (p. 42), Schön (1983) refers to this process as “an irreducible element of art” in professional practice (p. 18).

How can we describe and conceptualize such an art of living, or working, with technology? I will trace one such process based on observations of how homecare workers engaged with new tele-monitoring technology. I focus on the ethical work—or art—caregivers displayed around one particular value challenged by the new technology: privacy, as it pertained to the lives of their clients. Borrowing the term from Pols (2012), I call this art “modest” in the sense that it takes place, quietly as it were, within mundane activity.

## Tele-Monitoring and Privacy in Dutch Homecare

The tele-monitoring system discussed here employs motion sensors, placed in the homes of frail elderly people to track daily patterns of sleeping, eating, toileting and general activity. These patterns are scanned for acute or gradual changes in activity that could indicate emerging problems with health and well-being. Because the system stores and compares data, it can track gradual changes in activity not easily noticed by human eyes. The information is made available to the co-ordinating caregivers for particular clients through a password protected website. Significant changes are sent as SMS alerts to their cellphones. Activity monitoring offers caregivers information about their clients beyond direct observations and talks during care visits. Homecare organizations introduce this technology to improve the diagnosis of their clients’ health and actual ability for self-care. On this basis they hope to support vulnerable elderly people to live at home longer.

My research took place between September 2010 and January 2013 and started when activity monitoring technology was introduced with three homecare organizations in the south of the Netherlands. I attended meetings in which caregivers were trained in using the technology and follow up meetings in the 9 months afterwards in which teams of caregivers discussed working with the system. I interviewed five caregivers more in depth about their experiences, and spoke with managers and trainers of similar projects in the Netherlands and Belgium.

The introduction of new technology, especially when this takes place top-down as in the case observed, often starts with confusion. Normal routines are partly broken and the implicated knowledge and values get challenged. At the instruction meetings I attended, caregivers had many questions about how to fit the technology into their daily routines. They were also specifically disturbed about the privacy issues that activity monitoring raised for their clients and wondered openly whether the system was acceptable. To these last remarks, trainers and managers responded that caregivers would come to see the advantages of enhanced monitoring in working with the system, suggesting these advantages weighed up to their concerns. However, this is not exactly what I observed in the months that followed.

After the introductory meetings, caregivers started to experiment with the system. Though many, often to their own surprise, indeed came to like working with it, concerns for their clients’ privacy did not simply evaporate. Instead caregivers designed ways of fitting the technology into the relationship with their clients that would respect their clients’ privacy. In this process, what privacy means in the new technologically mediated situation was re-articulated.

Discussing the current widespread public debate on privacy from the perspective of law- and policymaking, Solove (2008) remarks how the word “privacy” is used with great emotive force, while no one can state clearly what it means. This was not different in the team meetings. Despite their worries, caregivers could not really name the “privacy issues” involved. This is not strange when we consider, as Solove also suggests, that privacy in everyday practice means a multiplicity of things. It refers to bodies, homes and personal space, but also to public expressions about persons and to reputation. Privacy is at stake with the disclosure of data about people, such as information about sexual habits, income or shopping behavior, but also with specific uses of these data. Moreover, under the pressure of new information technologies that enable capturing and mining data on an unprecedented scale, the meaning of privacy is changing. New user practices on social media, in which people voluntarily publish vast amounts of personal data, also suggest that notions of privacy are in flux.

Next to the cultural disarray around privacy, two additional reasons explain why caregivers experienced difficulties articulating privacy issues they feared were at stake. Firstly, at this point it was still unclear what monitoring technology would actually mean in their daily work, except that it seemed to invite them to check on their clients’ behavior in ways that made them uncomfortable. Secondly, values normally do not exist as independent entities. They are articulated in specific acts and rarely in exactly the same way. To define a value unambiguously for all cases in which it appears at stake is difficult. Solove proposes to investigate the multiple meanings of privacy based on Wittgenstein’s notion of concepts as a family tied together by various resemblances. This probably suits not just for privacy, but most other values too.

However, in working with the technology, I would suggest, caregivers did articulate what privacy means. Mapping their activity, six more or less distinct meanings of privacy emerged.

### Privacy as Personal Dignity

Proper care is premised on the detection of vulnerabilities. However, clients do not always mention problems they face. They do not want to be a burden or are ashamed, specifically about problems with basic bodily functions. Caregivers discovered how monitoring technology could help to relieve their clients from having to openly mention shameful problems and ask for help. Because caregivers could observe irregular patterns of sleeping, eating or toileting, they could initiate discussion on problems with diarrhea, with pain or anxiety in the night, or with managing meals. The system, they found, at least partly also supported clients’ privacy by speaking for them about undignified vulnerabilities.

### Privacy as Psychic Space

Clients give permission to install the system, but tend to forget the presence of the sensors after a while. When caregivers received data, they had to decide whether and how to communicate these to clients. However, alerts or data did not always prove meaningful. The sensors could indicate declining meal preparation, while caregivers knew their client was eating out. Alerts on nighttime activity could signal pain, disturbed sugar levels or beginning dementia, but sometimes being a nighthawk simply belonged to a person. Caregivers found they needed to interpret data with their client in mind. They opted to ignore data they deemed meaningless and would not mention these to clients. To remind clients of “being watched” when it made no difference in the care for them, they discovered, burdened clients and made them “feel watched” unnecessarily.

### Privacy as Personal Perspective

Caregivers were sometimes confronted with situations where the technology indicated something different than what clients communicated. Clients would minimize troubles or conversely, complain about a bad sleep, while the system suggested the opposite. When problems didn’t seem serious, caregivers found they “shouldn’t press on”. Clients, they pointed out, are entitled to their own views and their own motives for what they tell caregivers. As one caregiver put it, “She doesn’t have to let me in on everything.” Caregivers also learned that discrepancies could signal other needs than those phrased, such as loneliness or anxiety. Instead of keeping distance to leave their clients’ personal perspective in place, these cases needed extra attention to bring that perspective out.

### Privacy as Control Over One’s Life

Some observations did need inquiries. Here, the solutions caregivers developed differed strongly. Those who were very uncomfortable with this task resorted to asking family or colleagues what they felt was going on. Others carefully explained their observations to clients, offering them room to formulate their views on how to meet needs. As one caregiver explained, clients need control over their care. Respecting privacy, another said, entails that “I make transparent what I see and share vulnerability and decision making”. Interestingly, she thereby indicated that caregivers have to give up some of their “privacy” by offering their observations up to scrutiny.

### Privacy as a Gift of Trust

Many caregivers felt that monitoring brought them closer to their clients, because they received a better picture of their clients’ personal rhythm of life, but also because their clients knew they made these observations and trusted them with these. They felt grateful to their clients for that.

### Privacy as Based on Ways of (Not) Looking

Caregivers indicated discomfort with data that gave “too much” information on their clients, particularly on the exact time and place of activities in the home. They ignored these, unless pressed by emergencies. They suggested it was important to not look at more than you need to know, or formulated differently, not to look for the sake of looking. Caregivers explained in interviews that observations are and should be part of their professional relationship with their client and not mere acts of curiosity.

In fitting activity monitoring technology into their daily practice, caregivers moved from concern and confusion about their clients’ privacy to pragmatic solutions of privacy problems. These solutions represent ethical work in re-articulating what privacy is, how it matters and what could be fitting norms to respect it. This process was accompanied by talk, in which caregivers referred to “privacy”, “personal space”, “feeling watched”, or more elusively to “Big Brother situations”, but they didn’t discuss privacy as such. They communicated experiences and solutions. Re-articulating privacy took place mostly in-action. I will now discuss some structural characteristics of this process, drawing on the exploratory work of Schön (1983) on reflection-in-action, supplemented by some suggestions derived from Lyotard’s (1988, 1991) rethinking of Kant’s aesthetic reflection.^1^

## The Art of Reflection-in-Action

Schön (1983) defines the artistry of professionals against mistaken conceptions of professionalism as the instrumental solving of standard problems, based on acquired specialized knowledge. He argues that practice is too unstable for such a “technological fix” (p. 15) and provides an alternative epistemology of practice in which reflection-in-action is central. Reflection-in-action is the capability of professionals to engage with complex, indeterminate situations that challenge their tacit knowledge and former experience within their practice. These situations appear as “messes” (p. 18). A requirement for reflection-in-action and a first structural moment of the process is openness to “messes”, or, as Schön puts it more positively, to situations in their uniqueness. Being open is not being passive, simply letting messes wash over one. It is an active attitude. Lyotard (1988) speaks about “passibility” (*passibilité*) (pp. 121, 190), the capacity to suspend business as usual and put established assumptions on hold in order to “receive” new possibilities.

In the case discussed, although management introduced the technology, working with it started as a pilot case on a voluntary basis. Nevertheless, caregivers still had to take the further step of actually committing themselves to engaging with the new technology and with the “mess”, in Schön’s sense, it seemed to entail for their clients’ privacy. Some caregivers drew back, not overtly, but by simply not using the technology. Others jumped in, which takes courage and some confidence in one’s ability to find a way out.

Engaging with situations in their uniqueness does not give one much hold. As explained earlier, even what the problem was, privacy in this case, could not be defined. Problems, Schön (1983) makes clear, have to be set. Yet, with existing assumptions challenged, this can only be done by moving ahead. In this process, experimental “moves” tentatively frame elements of the problem and suggest solutions, but these may need revision several times. Schön (1983) calls this a “reflective conversation” (p. 130) with the situation, in which professionals try out something, while listening to the talk-back of the situation. It entails a kind of double vision, keeping multiple possibilities open, and considering moves preliminary, while a trajectory towards a solution gradually forms.

Caregivers started to work with technology that they felt entailed serious privacy problems. In order to discover that the system did not just threaten their client’s privacy, but could also protect their personal dignity, caregivers partly reframed the way they intuitively saw the system at first. They stopped seeing it as only a tool for surveilling and checking on clients and started seeing it as a device that could also speak for their clients. Tacit ideas, that respecting someone’s privacy entails not observing them when they can’t look back, were suspended while a new sense of protecting someone’s dignity formed. This implied new obligations, such as becoming sensitive to data that could indicate shameful vulnerabilities clients might hide and finding tactful ways of opening conversation about these.

Another assumption caregivers had to let go was that data represent information to be had and used. Sometimes data were irrelevant or they were “too much” and had to be ignored. In the beginning, caregivers also often felt they had to be absolutely open to clients about observations in order to be respectful. This didn’t work well. Clients responded by getting disturbed and caregivers realized they needed to find ways of dealing with observations that didn’t make clients “feel watched” in ways that hindered them. Caregivers discovered that weighing what to watch or ignore, and what and how to communicate, while keeping individual clients and the relationship with them as a relationship of trust in mind, had to be their responsibility.

Conversing with the situation, caregivers gradually circumscribed the place the technology could and should take in the relationship with their clients and their own place in the new set-up. “The system should stand in the background” and “One shouldn’t insist on one’s watching role” were formulations of this. Protecting clients’ privacy where technology threatened it implied that they had to develop ways of watching data and ways of closing their eyes on them. All the while they did ethical work in re-articulating what privacy is in multiple ways and how it is to be cherished in the new technologically mediated situation. The framing of the many-sided problem of privacy happened while experimenting with the technology and finding solutions.

Though willingness to suspend assumptions and openness to new possibilities are vital to this process, not everything is in flux in reflection-in-action. Professionals draw on values that remain standing and also on former experience that they extend creatively into the new situation, “seeing” the last “as” a new example of their repertoire (Schön 1983, p. 139). Caregivers in the case study drew heavily on their experience with and sense for good relationships. They remained committed to solve privacy problems in ways that enabled their clients to flourish and that secured mutual trust.

In sum, reflection-in-action appears as a process in which openness to a situation in its uniqueness and willingness to suspend assumptions lead to an experimental conversation with the situation. In this process problems and solutions are articulated together by making moves, choosing among different possibilities. Moves are subsequently either abandoned or pursued further, while a trajectory to a solution forms. How can this process be seen as artistic or aesthetic?

Lyotard (1991) makes a distinction between two aspects of aesthetic reflexivity as a faculty of the mind that can help to clarify the nature of this process, a “heuristic” and a “tautegoric” one (p. 16). The first refers to the capacity to move beyond what one knows, based on hunches of how or what things might be. Keeping multiple possibilities open, trying out things in an as-if and what-if mode form the core of this imaginative capacity. According to Lyotard (1991) the heuristic faculty functions in art in its most free or “naked” (p. 19) state. In science, as in professional life, creativity is constrained by specific goals, such as providing good care, and by practices as they exist. While an artist may strive to shake off all conventions to begin anew, professionals keep part of their acquired “attire” in place and bring more of their former experience to bear on the new. Pols’ term “modest” to distinguish a practical, professional aesthetics from the more grandiose creativity and beauty of art seems quite suitable here. The difference is one of degree and of context.

The second aspect of aesthetic reflexivity, tautegory, designates what orients creative heuristic moves. It is the ability of the mind to receive impressions and to feel itself at the same time in relationship to these, and to take this feeling as its guide. Lyotard compares it to the proprioceptive sense of left and right that orients the human body in space. In its basic state it is a feeling of pleasure or displeasure, of harmony between the mind and what it senses, or of difference. Next to the creative nature of the process, what makes reflection-in-action artistic or aesthetic is this orientation on feeling.

While the improvisational nature of what caregivers did became clear above, the tautegoric aspect of their activity can now be elucidated. Caregivers referred a lot to feelings, their own, such as concern, resistance or feeling right about something, but also their clients’ feeling of flourishing, being cared for, or of feeling psychically confined by watching eyes. They received their clients’ trust with gratitude and a new sense of connection. More importantly, they sounded out the proper place for the technological system and their own position towards the data aesthetically. They indeed attempted, as Pols (2012) argues, to bring about “fits” and avoid “misfits” (p. 43). Both fits and misfits are sensed as a relationship in the world “between relevant variables” and tautegorically in the relationship of caregivers to these, as feeling uncomfortable, hopeful or right about something. What enabled working with the technology was finding proper balances between, for instance, not seeing enough and seeing too much.

While a feeling for fits, balance or harmony resonates with the aesthetics of beauty, looking for fits begins in a feeling closer to the sublime, the aesthetic fascination for something incomprehensible that carries the mind beyond the familiar. In this experience, disturbance about what appears as chaotic goes together with a feeling of commitment to unknown possibilities. Commitment to the unknown returns during the process of reflection where professionals don’t remain within their comfort zone, but take risks in finding new and as yet unimagined fits. Lastly, a “modest” experience of the sublime, as a feeling for what does not fit with the mind but transcends it, either temporarily or more fundamentally, is vital to the whole process of reflection-in-action. It is an awareness of the always tentative nature of human acts and knowledge. Openness asks courage and confidence, but also entails humility.

## Closing Remarks: An Art of Relating

Ethical engagement with technomoral change, instigated by new technologies, I hope to have shown, does not just take place as overt deliberation and argument. It is also done reflecting in-action, in the midst of daily practice. I have presented reflection-in-action as a “modest” artistic activity, proceeding creatively on the basis of tautegoric feeling, within a given professional practice. The significance of processes like these goes beyond care and professional practice more generally, and warrants closer investigation in various everyday situations, as part of an art of living with technology.

Whether engaged with technomoral change, or other changes evoked by technology, such an art is an ethical project in itself. Verbeek (2011) presents it as a self-practice, a more or less conscious styling of one’s subjectivity in relationship to technology. I will close my argument by proposing another, more “modest” alternative. Firstly, this art originates in humans as relational beings, whose identity is formed in relationship to the beings around them, whether human, animal or material ones—including technological beings. Secondly, it is oriented towards establishing good relationships.

Caregivers reflecting on privacy problems in the relationship with their clients were not aiming to style their own subjectivity and life as a work of art in relationship to new technology. They were securing good relationships. This process was guided by caregivers using their feeling and aesthetic sense, including moral intimations of what they felt was right, but to see this as a project of and for a self does not do justice to it. I chose a specific moral conflict as case here. While morality is relational par excellence, having to do with what human beings owe each other and with visions of a good human life (Swierstra 2013), the art caregivers displayed was also relational in a more general sense. The situation they found themselves in, together with their clients, was co-shaped by technology. As Verbeek (2011) contends, humans and technologies co-constitute each other. Moreover, openness to messes and trying to find a way out entails that one allows oneself, one’s knowledge and values, to be changed by occurrences. Oriented by feeling and sounding out relationships in the world, reflection-in-action is not the capacity of a self or subject, positioned towards a world as object. It is an art of relating, belonging to relational beings, and oriented to bringing about good relationships in the world of which one is part. Instead of implicitly borrowing from theories that see art as the activity of individuals, or creating one’s life as a work of genius, theorizing an art of living with technology could gain from a more “relational aesthetics” (cf. Bourriaud 1998) that sees artworks as events and sets of relationships with the world, and their creators as operators within these.

Interestingly, a task such as the one Bourriaud assigns to contemporary art, to strengthen conviviality and turn technologies from economic into life possibilities, places the activity of caregivers discussed here at the forefront of an artistic project. Articulating the multiple meanings of privacy in technologically mediated care, caregivers devised solutions that are of value beyond the context of healthcare. They demonstrated that attitudes towards personal data, ways of looking at them and ignoring them, as well as a sense for data as a gift to be cherished, are as important as setting material and legal limits to technological possibilities. In this, they point to vital human relational capital in finding solutions to problems that technologies introduce. This can be a significant contribution to moral innovation in the age of extended data mining and social media, in which ways of (not) looking and of “valuing” data could be further developed as part of civil life. The art of living with technology explicitly shows itself here as an art of living with technology *together*.

## Footnotes

1 For an elaboration on fruitful similarities in Schön and Lyotard on the topic of reflection, see Kamphof (2004).
